# Community pharmacists’ perceptions and experiences of stress during COVID-19

**DOI:** 10.1186/s40545-023-00523-6

**Published:** 2023-01-27

**Authors:** Loreta Tobia, Mario Muselli, Fabio De Luca, Vincenza Cofini, Giada Mastrangeli, Leila Fabiani, Stefano Necozione

**Affiliations:** Department of Life, Health and Environmental Sciences, University of L’Aquila, (MeSVA-UNIVAQ), Piazzale Salvatore Tommasi 1, 67100 Coppito, AQ Italy

**Keywords:** Community pharmacists, Work, stress perceived, SARS-CoV-2, COVID-19

## Abstract

**Background:**

Pharmacists play a crucial role in the COVID-19 pandemic scenario, performing frontline roles for the community, and supporting the healthcare system. This study aimed at investigating stress and its correlates among this category of workers at a high risk of SARS-CoV-2 infection.

**Methods:**

The participants for this study were employees of the “Municipal Pharmaceutical Company” of L’Aquila (Italy). Data were collected using an anonymous, web-based, self-administered questionnaire. Two independent surveys were conducted, from June to July 2020, and in January 2021.

**Results:**

Two separate groups of respondents were involved: a total of 37 workers participated in the first survey (mean age 44.9 ± 10.7, 75.7% women) and 18 in the second survey (mean age 45.9 ± 9.2, 94.4% women). The average total scores of the perceived stress (GHQ-12 score) increased significantly from 15.5 to 18.2 (*p* = 0.0438), showing a moderate level of stress in the category investigated.

**Conclusions:**

We observed a strong emotional exhaustion in the study sample of pharmacists, who reported high-risk perceptions and fear. A comprehensive assistance should be granted to support the well-being of healthcare workers who provide an essential service, despite the high risk of infection.

## Background

Italy was one of the most affected countries in the early stages of the pandemic: it was the first European country to face the SARS-CoV-2 spread with 1,651,229 confirmed cases and 57,647 COVID-19 attributed deaths between February and November 2020 [1].

Following the public health emergency of international concern by the World Health Organization on 30 January 2020, several measures were adopted by the government to manage the spread of SARS-CoV-2 in the community and in places of work [2].

In particular, Italy implemented the "Shared protocol regulating the measures to contain the spread of the COVID-19 virus in the workplace" which was updated in April 2021 and was renewed in April 2022 [3]. The document contains guidelines shared between the various social partners, companies, and trade unions, to help companies in the adoption of coronavirus preventive and protective measures and safety protocols in the workplace. In 2020, the Italian National Institute for Work Insurance (INAIL) implemented a methodological approach adapted from a model developed on the O'NET database of the US Bureau of Labor of Statistics [4] to estimate the occupational risk of infection, classifying each economic sector as at low, medium–low, medium–high and high risk, based on three parameters: exposure probability, proximity index, and aggregation factor [4]. Pharmacies and personnel have been classified as being at high risk of SARS-CoV-2 infection like the whole health care sector, due to the probability factor and contact with potential infected people.

Pharmacies were the first point of contact for healthcare provision and have historically played major roles during pandemics and viral outbreaks. Pharmacies provided different services to support the national health system during the COVID-19 pandemic: delivering drugs to patients, educating patients about telehealth services, evaluating patients for the renewal of prescriptions of chronic medication, consulting on minor ailments, dispelling misconceptions about COVID-19 treatments, contributing to COVID-19 screening, and more. The spread of COVID-19 has caused unprecedented psychological stress among health care workers worldwide, resulting in the risk of anxiety, depression, insomnia, work-related stress, and post-traumatic stress disorder [5, 6]. Emotional distress is mainly associated with the perception of risk, the countless deaths, the perception of the uncontrollable risk, long work shifts, and working directly with positive COVID-19 patients [7].

Pharmacists have always been among the most accessible providers of care services, This is especially true in the era of COVID-19. Community pharmacies remained open to the public despite the stricter restrictions. They contributed to the management protocols of COVID-19, and played a crucial role in the administration of COVID-19 vaccines to achieve a broad vaccination coverage in a reasonable time. Furthermore, by providing screening and testing activities for patients, pharmacies have contributed to flattening the contagion curve. Pharmacists have demonstrated good levels of knowledge of COVID-19 and have shown a high-risk perception [8]. Recent studies have shown that pharmacists have burnout and secondary traumatic stress (STS) rates like other healthcare professionals: 47% of pharmacists reported developing a burnout syndrome, and 51% of that burnout was related to the COVID-19 pandemic [9]. Although many studies have been conducted on stress perceived among healthcare professionals, to the best of our knowledge, there are few studies regarding stress among pharmacists and community pharmacy workers, classified at high risk of infection due to the proximity to potential infected people. We therefore decided to contribute with a study aimed at investigating perceived stress and its correlates. Better knowledge of the factors related to work stress during a pandemic could help to better understand work-related stress and provide useful information to implement corrective actions for health care providers.

## Methods

### Study design

This study was a comparison of two surveys conducted on the employees of the “Municipal Pharmaceutical Company” in the town of L'Aquila, in Abruzzo region, Central Italy. Community pharmacists of the city of L’Aquila are a small group of healthcare professionals. The study was authorized by the Internal Review Board of the University of L’Aquila as part of the project “Knowledge, attitudes, perception of the risk of COVID-19 infection in university students and workers with different degrees of risk” (IRB: n. 31/2020).

### Data collection

Employees were contacted and invited to fill in an anonymous, web-based self-administered questionnaire after they gave their consent. Because of the high turnover in staff, it was impossible to administer the second survey to the same workers. In fact, many pharmacists left their positions and were replaced by others. Therefore, the study is intended as a comparison between two independent surveys.

The questionnaire included 5 sections:

Socio-demographic information (sex, age, cohabiting, number of cohabiting), work information (degree and working age), adherence to flu vaccination, and smoking.

The General Health Questionnaire (GHQ-12): GHQ-12 is a 12-item self-reporting instrument for the detection of mental disorders in the community and in non-psychiatric clinical settings [10].

It measures aspects of anxiety, depression, and social functioning. Although it does not yield a diagnosis, positive scores are indicative of psychological distress. The GHQ-12 asks respondents to report how they have been feeling over the past few weeks using a 4-point scale (“more than usual, as usual, less than usual, and much less than usual”). It is scored using a numerical  response format (Likert scale: 0–1-2–3), resulting in a scale ranging from 0 to 36. A score of < 15 indicates normal levels of stress, a score between 15 and 20 indicates the presence of stress, and a score of > 20 indicates severe psychological distress. In a community study, the sensitivity and specificity in predicting cases of psychiatric morbidity were 69,6% and 94,8%, respectively [11].

Modified items from the "Standard questionnaire on risk perception of an infectious disease outbreak” [12], with close-ended questions based on 5-point Likert scale and binary questions (yes/no), about the various aspects of COVID-19 were used to evaluate different topics:

*Knowledge items* The purpose is to measure the level of knowledge of the respondents about the various aspects of the disease (such as infectiousness, fatality, transmission route or preventive/control measures); the score varies from 0 to 20. As an emerging infectious disease, we assumed that knowledge about the causing organism, the pathophysiology of COVID-19, and the effectiveness of public health treatments would increase quickly; therefore, we projected that this would have an influence on respondents' stress.

*Perception of self-efficacy* These items provide an estimate of whether the respondents thinks they can adhere to the preventive measures; the score varies from 11 to 33.

*Perception of susceptibility to the disease* The items regarding risk perception focus on the individual chances of contracting the disease during a certain period; the score varies from 0 to 20.

The measure of self-perceived health and quality of life (HRQoL) used by the Italian Surveillance System “PASSI (Progress of the Health Authorities for Health in Italy” [13, 14]) was developed by the Centers for Disease Control and Prevention [15, 16], and it is based on the answers to the following questions:

- How is your health in general? (Excellent—very good—good—fair—poor).
- Now, thinking about your physical health, which includes physical illness and injury, for how many days in the last 30 days was your physical health not good?
- Now, thinking about your mental health, which includes stress, depression, and problems with emotions, how many days during the past 30 days was your mental health not good?
- Now, thinking about your usual activities. In the last 30 days, for about how many days did poor physical or mental health keep you from doing your usual activities, such as self-care, work, or recreation?

The two HRQOL questions on physical and mental health were summed to calculate the “Summary Unhealthy Days Index”, with a maximum of 30 days. The total number of unhealthy days was calculated as the sum of days in poor physical health and that in poor mental health in the last 30 days up to a maximum of 30. It is a validated index of self-reported mental and physical health that allows researchers to examine trends in health over time and identify groups of people that may need attention [17].

Data were collected from June to July 2020 after the Italian lockdown, and in January 2021, during the second wave of the COVID-19 epidemic in Italy. We decided to administer the second questionnaire during the pandemic second wave in Italy in order to evaluate workers’ psychological response in a period when there was a better knowledge of the SARS-Cov-2 infection and stronger preventive measures and protocols were applied.

### Statistical analysis

The first outcome was the estimation of the perceived quality of life and its relationship with the demographic factors, training, perceived stress, and perception of susceptibility to the SARS-CoV-2. All variables were analyzed and reported as frequencies or mean and standard deviation (SD) or median and interquartile range (IQR), if they were qualitative or quantitative variables, respectively. The analysis was carried out with the STATA 17 software, setting alpha to 0.05. Statistical comparisons were performed using either the Student’s *T*-test or the Mann–Whitney *U* test, as appropriate. The Chi-squared test or Fisher exact test was used to compare the equality of proportions between pharmacists' groups. To estimate the factors associated with perceived stress (GHQ-12 score), we conducted a multivariable analysis including the following variables: time (first and second survey), sex (female and male), age, degree (graduate and undergraduate), knowledge score, preventive measures, risk perception, unhealthy days index, and adherence to flu vaccination.

## Results

All workers responded to the two surveys. A total of 37 workers participated in the first survey, while only 18 workers participated in the second survey. Table 1 shows that the participants in the second survey were older and predominantly female. There was a significant difference between the surveys with respect to flu vaccination: the proportion of vaccinated workers increased from 8.1 to 33.3% (*p* = 0.046).

**Table 1:** Participant’s characteristics at the 1st and 2nd survey

Characteristics	1st survey		2nd survey		*p*-value
	Mean or *n*	SD (*%*)	Mean or *n*	SD (*%*)	
Gender					
Male	9	24.3%	1	5.6%	0.090
Female	28	75.7%	17	94.4%	
Age	44.9	± 10.7	45.9	9.2	0.634
Cohabiting					
Yes	26	70.3%	15	83.3%	0.456
No	11	29.7%	3	16.7%	
Number of cohabiting					
0	10	29.7%	3	16.7%	0.504
1	8	21.6%	7	38.9%	
2	8	21.6%	4	22.2%	
3 or +	11	27.1%	4	22.2%	
Degree					
Graduate	29	78.4%	15	83.3%	0.757
Undergraduate	8	21.6%	3	16.7%	
Years of work	19.7	± 10.7	18	± 7.9	0.720
Flu vaccination					
Yes	3	8.1%	6	33.3%	0.018
No	34	91.9%	12	66.7%	
Smoking					
Yes	6	16.2%	2	11.1%	0.614
No	31	83.8%	16	88.9%	
Knowledge score	11.9	± 3.1	14.9	± 2.1	< 0.001
Adherence to preventive measures	31.5	± 2.0	31.1	± 2.2	0.533
Risk perception	12.4	± 2.3	12.7	± 2.1	0.750
Unhealthy days	3.3	± 6.5	8.1	± 8.9	0.006

The knowledge score showed a statistically significant increase between the first and second survey, from an average of 12.1 to 14.9 (*p* < 0.001). The score on the perception of disease risk showed a high level in both the first and the second survey: in the first survey the average score was 12.3 out of 16 while in the second survey was 12.7 (not statistically different, *p* = 0.624). Preventive measures score showed high values since the first survey. In fact, in the first survey the average score was 31.3 out of 33, overlapping the average score of 31.1 obtained in the second survey (not statistically significant).

The total number of unhealthy days increased statistically significantly during the second survey (*p* < 0.0001) from an average of 2.9 (± 5.9) to an average of 8 (± 8.9).

**Fig. 1:**
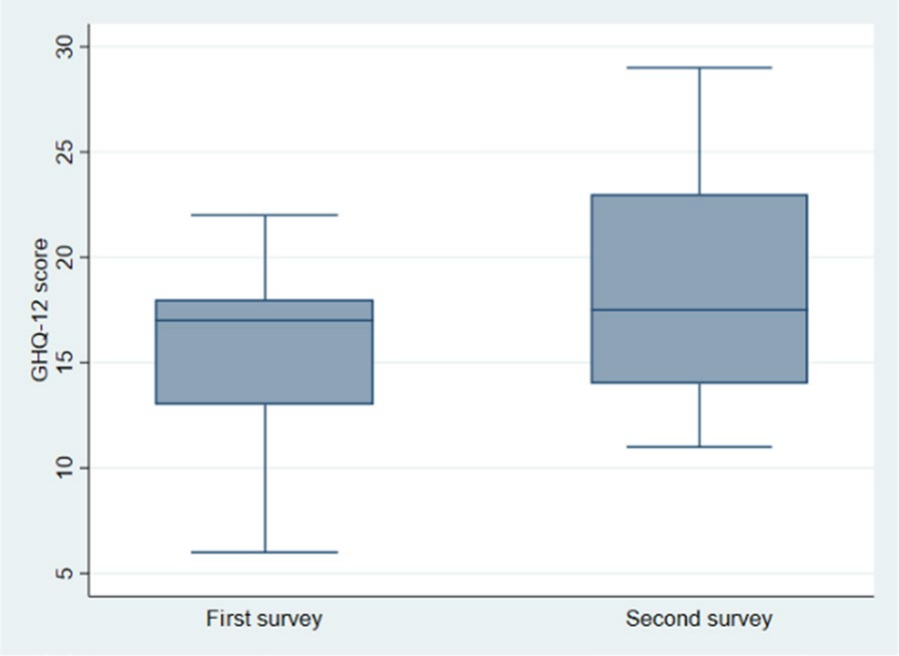
GHQ-12 scores in first and second survey

The average total scores of the perceived stress (GHQ-12 score) did not increase significantly between the two surveys: from 15.7 to 18.4 (*p* = 0.169), showing a moderate level of stress among the workers during both the surveys (Fig. 1).

We built two regression models, one for each survey, to identify possible factors related to the GHQ-12 score as a proxy of the stress perceived by workers. The factors considered were age, sex, degree, years of work, knowledge score, adherence to preventive measures, adherence to flu vaccination, risk perception and unhealthy days index. In the first survey, the multivariable analysis showed that no factors were related to GHQ-12 score (*R*^2^ = 0.3113; *p* = 0.3799). As shown in Table 2, the multivariable regression model built for the second survey (*R*^2^ = 0.8947; *p* = 0.0045) identified flu vaccination and unhealthy days index as factors associated with GHQ-12 score. Psychological distress increased as the unhealthy days index increased and among workers who carried out flu vaccination.

**Table 2:** Multivariable regression model second survey

Characteristics	Coefficient	Confidence interval (95%)		*p*-value
Age	− 0.533	− 1.469	0.402	0.229
Degree				
Graduate	Ref			
Undergraduate	− 2.274	− 9.374	4.825	0.487
Years of work	0.901	− 0.240	2.041	0.108
Knowledge score	0.563	− 0.702	1.829	0.340
Adherence to preventive measures	0.124	− 0.838	1.085	0.778
Flu vaccination—yes	6.954	3.074		0.003
Risk perception	− 1.005	− 2.596	10.835	0.187
Unhealthy days index	0.284	0.078	0.490	0.012

## Discussion

Pandemics are known to generate anxiety, depressive disorders, or post-traumatic stress among hospital-based health care workers [18]. However, the effects on other workers in essential activities, such as pharmacies, that have also been exposed to people who may be infected, have been less studied. In fact, community pharmacies represent an essential service during a pandemic lockdown, not only to ensure an adequate supply of medicines, but also as a reference service for patients seeking information and education on COVID-19 and the related aspects. We have found that among community pharmacists, COVID-19 has caused a worsening of the perceived stress, despite their adequate knowledge of the disease and effective preventive measures.

The first survey was conducted after the national lockdown, when the virus pressure on the population decreased. The survey reported that 64.9% of study participants experienced psychological distress (GHQ-12 ≥ 15): this result is similar to that obtained by Tahara et al. in the same period 8 [19]. However, we found lower levels of mental stress than those detected by Buonprisco et al. on Italian community and hospitals pharmacists [20].

Another study conducted at the University of L’Aquila (Italy) on 130 rehabilitation providers working in Italian centers for pediatric neurocognitive, speech and psychomotor rehabilitation, showed that the rehabilitation providers with moderate or severe stress level were more likely to have a negative perception of the quality of life [21]. Also, medical school students who were forced to distance on line learning during pandemic suffered from psychological and physical stress [22].

Previous studies conducted in the pre-COVID-19 pandemic among Italian healthcare workers after the 2009 L’Aquila Earthquake reported that the prevalence of “psychological distress (GHQ-12 ≥ 15)” was 52.5% [23, 24]. A meta-analysis reported prevalence rates of 23.2% for anxiety and 22.8% for depression among healthcare workers during the COVID-19 pandemic [25]. Another study reported that stress levels among medical staff during the pandemic were higher than usual [26]. Our results indicate that in Italy, as in other countries, the implementation of COVID infection control measures has had a substantial mental health impact on healthcare workers after had been realized [27, 28].

Rates of mental health problems were previously reported to be higher among healthcare workers than among the general population [29]. Approximately 40.4% of the general population was reported to have psychological problems resulting from stress associated with COVID-19, which was lower than the 64.9% rate in our study participants [30]. Our findings confirm that healthcare professionals experienced serious mental health issues during the COVID-19 pandemic, emphasizing the need for action. In contrast to other studies [19, 31], our results showed no gender gap, with males and females having the same prevalence of psychological stress. We have observed a substantial increase in the score related to knowledge across the two surveys: this shows the importance of staying informed among healthcare workers.

We found a significant increase in the knowledge score across the two surveys; however, there were no differences in the perceived risk of the disease and the adherence to preventive measure. On the one hand, these findings seem to suggest a remarkable ability to stay informed on the advances in the knowledge of the disease; on the other hand, the findings seem to suggest the ability to comply with the appropriate and recommended preventive measures.

Nevertheless, with respect to the Summary Unhealthy Days Index, our study reported an average of 3.3 unhealthy days at first survey, and 8.1 at second survey, higher than the 4.4 days reported by the PASSI system pre-COVID. This significant difference can be explained by the different detection times in relation to the pandemic trend: the recovery from the disease, also associated with the emergence of new variants, had a negative impact on the health status perceived by this group of workers.

We found a different proportion in flu vaccinations across the two surveys: fear of SARS-CoV-2 infection might have prompted many workers to get vaccinated in order to increase their safety.

Indeed, the multivariable analysis revealed that during the second survey, psychological distress was significantly related to the Summary Unhealthy Days Index and adherence to the flu vaccine. The need to ensure an essential service despite the higher risk of exposure could have been an important stressor in the complex health environment during the pandemic. Subjects who had a higher level of psychological distress and a worse state of health may have tried to protect themselves by using flu vaccination.

Our study presents some limitations. The study was limited to community pharmacists, so the results are not reflective of trends in the general population. Moreover, the sample was unbalanced with regard to work category and gender, with a higher presence of women: however, this different gender distribution reflects the Italian national trend where a greater proportion of females are employed in community pharmacies.

Other limitations were mainly related to the dropout rate and the lack of psychological evaluation of the participants and their coping strategies. As known, the problem of the dropouts in longitudinal surveys may be related to three separate sources: failure to locate research participants, failure to contact participants, and failure to achieve cooperation. The use of the online questionnaire due the COVID-19 epidemic could be another factor related to the high dropout rate. However, staff turnover should be considered: 19 workers terminated the employment relationship between the two surveys. Despite this, the characteristics of the participants between the two surveys were overlapping.

Finally, the surveys were carried out at different times during the pandemic; while this may constitute a limit to the direct comparison of the results, on the other hand, it can identify changes in the behavior and health of workers exposed to different risks.

## Conclusion

No previous research measured psychological distress and the HRQoL among community pharmacists during a pandemic, and more research is needed to understand how to help these categories to better manage work-related stress during an emergency disease and its impact on their life. This study can provide useful information to the critical issues faced in conducting a fundamental survey on the assessment of the health status of workers.

In conclusion, our findings suggest that during the SARS-CoV-2 pandemic, pharmaceutical professionals have been subjected to significant emotional stress and feelings of great risk and fear for their own health and of their families. We suggested that a comprehensive assistance should be provided to support the well-being of healthcare workers especially those who continued to provide an essential service despite the high risk of infection.

## Data Availability

All relevant data and materials are within the paper and its Supporting Information files.
